# Editorial: Human digital twins for medical and product engineering

**DOI:** 10.3389/fbioe.2024.1489975

**Published:** 2024-10-16

**Authors:** Jörg Miehling, Julie Choisne, Anne D. Koelewijn

**Affiliations:** ^1^ Engineering Design, Department of Mechanical Engineering, Friedrich-Alexander-Universität Erlangen-Nürnberg, Erlangen, Germany; ^2^ Auckland Bioengineering Institute, The University of Auckland, Auckland, New Zealand; ^3^ Chair of Autonomous Systems and Mechatronics, Department of Electrical Engineering, Friedrich-Alexander-Universität Erlangen-Nürnberg, Erlangen, Germany

**Keywords:** biomechanics, personalized musculoskeletal modelling and simulation, human-technology interaction, sensorization, state estimation

## 1 Introduction

The current trend towards digitalization of human-centred engineering processes in conjunction with advances in (bio-) mechanistic modelling, high-performance computing, artificial intelligence (AI) and sensor technology leads to unprecedented transformation potentials in medical, product and human factors engineering. These advancements are significantly enhancing human-technology interaction and improving medical treatment outcomes. Biomechanical simulations hold high potential by revealing the processes and inner strain conditions of the human body. For reliable simulation results, a suitable model is required, as well as a measurement, estimation, or prediction approach to analyse human motion behaviour, its interaction with the environment and, if present, its interaction with technology. In this context, we refer to a human digital twin as a virtual representation or digital replica of an individual, created using data from various sources, including sensors, medical records, and other digital inputs. This digital twin mirrors certain physical and behavioural characteristics of the person, enabling simulations, predictions, and analyses. If interactions between humans and technology are considered, the concept of digital twin couples is applicable. This concept facilitates the use of human digital twins and digital product twins (within its environment) in conjunction with different data streams, which can be measured on the human, on the product, or at their interface. The combined use of different data streams may enable a more accurate estimation of the states within the overall human-technology system in terms of a model-based systems engineering approach. This subsequently allows to optimize physical human-technology interactions based on simulations, estimations, or predictions with the digital twin couples as well as data transfer between virtual and physical instances of human and technology.

The goal of this Research Topic was to explore human digital twins as personalized biomechanical models for person-specific simulations and their application in human-centred engineering. Such simulations allow for computing biomechanical variables from wearable or unobtrusive sensors instead of requiring expensive gold standard lab-based equipment. Portable equipment and automatic processes lead to easily accessible biomechanical analysis for clinical applications, to permanent monitoring of usage scenarios, or to direct use of simulation-based information during the interaction between the human and a product. Digital product twins may be included, if observation, simulation or prediction of product behaviour is necessary in the prospective human-technology interaction use case.

Current biomechanical models are often generic and only scaled based on full bodyweight and marker data. Personalised medicine allows to customize biomechanical models to the patient-specific information allowing for more accurate prediction. For example, statistical shape models are replacing musculoskeletal generic models. Moreover, crucial factors such as muscle strength or range of motion are usually not personalized in a scaling-based generic model. In-depth patient-specific modelling from medical image data is often implemented for limited body regions, for which such data is available. Medical imaging is usually indicated for conditions that cause larger bone deformities, such as in cerebral palsy or for orthopaedic surgery planning. However, when medical imaging and standard motion capture data are unavailable, alternative methods for personalized modelling are needed.

## 2 Tackling the challenges in the design, implementation and application of human digital twin technology

The Research Topic Human Digital Twins for Medical and Product Engineering encompasses a broad overview of this research area through eleven excellent contributions which include one systematic review, one brief research report and nine original research articles. These theoretical, computational and experimental studies can be grouped into the following focus areas of the Research Topic.

### 2.1 Approaches towards motion data processing with digital human models

The systematic review by Wechsler et al. provides a comprehensive overview and recommendations for minimizing the sim2real gap – the discrepancy that commonly exists between musculoskeletal simulation outputs and real-world observations. This information supports those researchers using musculoskeletal models in conjunction with movement measurement data for both medical and product engineering applications. Moghadam et al. investigated the feasibility of predicting joint kinematics and kinetics with IMU sensors in conjunction with personalized machine learning models. This study proposes a promising personalized approach for gait time series prediction in children, involving an RF model and two IMUs on the feet. Michaud et al. created digital twins for patellar tracking and treatment prediction. This study considers two different approaches: the use of 3D models and contact detection algorithms. Eventually, simulation results were compared with experimental measurements from a sensorized 3D-printed test bench under pathological and treatment scenarios.

### 2.2 Parameter identification and modelling procedures for detailed person-specific modelling and simulation

Zot et al. studied the mechanical effects of lumbar disc arthroplasty on the facet joints by creating patient-specific finite element models of the intact and post-arthroplasty lumbar spine based on CT-scans of lumbar spine specimens. These models have been applied to compare the mechanical response of both ball-and-socket and elastic protheses under physiological loadings. Remus et al. conducted experimental human abdominal *in vivo* macro-indenter measurements to derive soft tissue material properties based on time-of-flight sensors for 3D displacement measurement of the body hull and surface electromyography (sEMG) to monitor muscle activation levels. Inverse finite element analysis was used to approximate the nonlinear material parameters of the soft tissue. Baier et al. simulated functional electric stimulation (FES) protocols on the forearm with muscle-specific activation resolution with a human digital twin consisting of an anatomically based 3D volume conductor, muscle specific nerve fibre arrangement and a specific nerve model. This approach can eventually be applied to determine the optimal procedure for neurological rehabilitation. Ortiz-Puerta et al. conducted a morphometric study of the proximal airways based on geometrical measures associated with the different airway generations. Accurate representation and characterization of the airway luminal surface and volume was informed by CT images of the respiratory tree and was applied to compare smoking pre-COPD and COPD individuals.

### 2.3 Evaluation criteria and test procedures

Nölle et al. investigated tendon strains in jersey finger load cases using a finite element neuromuscular model in conjunction with a newly defined injury criterion. Jersey finger injury occurs through an eccentric overextension of the distal interphalangeal joint leading to an avulsion of the connected FDP tendon. Achour et al. developed a biomechanical test bench for investigation of implant failure of different osteosynthesis systems on the mandible.

### 2.4 Predictive approaches

Van der Kruk and Geijtenbeek investigated the effects of muscle weakness and pain avoidance in individuals with unilateral knee pain on trunk flexion while standing up using a predictive neuromuscular simulation study. Killen et al. investigated *in silico* techniques to facilitate optimal personalized prescription of shoe insoles based on measured motion capture, inverse musculoskeletal modelling and forward dynamic simulation to predict the kinematic adaption to specific insole designs. The approach was subsequently used to study healthy participants and flatfoot patients.

## 3 Conclusion and future perspectives

The published articles provide valuable contributions towards personalized biomechanical modelling and simulation including model parameter identification, novel sensor data processing, and state prediction and evaluation based on musculoskeletal models. These results will gradually pave the way towards a productive applicability of human digital twins in the context of medical, product and human factors engineering. Nevertheless, some further aspects need to be researched in order to exploit the full potential of human digital twin application in bioengineering processes.

An ongoing challenge in biomechanics remains the identification of person-specific model parameters, such as muscle and material parameters for multibody and finite element simulations. Therefore, rethinking the paradigms concerning the choice of muscle and material parameters is of outstanding importance for the whole community (Nölle et al.; Remus et al.). On this basis, novel workflows need to prove clinical usability and the ability to optimize clinical outcome focussing on automated applicability, speed of use, use of more accessible data and possibly use of highly-accessible software for clinically applied human digital twin technology leading to effective and efficient patient-specific surgical and rehabilitation strategies (Zot et al.; Michaud et al.; Killen et al.; Baier et al.; Remus et al.). Generally spoken, simple and robust applicability is necessary for successful implementation also in non-clinical application areas. Predictive simulations offer valuable insights into the mechanisms behind altered movement strategies, potentially guiding more targeted treatment (Van der Kruk and Geijtenbeek; Killen et al.) and optimized human-technology systems. Moreover, multimodal approaches tracking kinematic and dynamic measurements may be one possible solution to handle the discrepancies between simulation results and real-world observations, reducing the sim2real gap (Wechsler et al.). In this context also novel experimental measurement setups and sensor technology are needed to accurately determine empirical reference values (Achour et al.; Remus et al.).

